# ClpX Is Essential and Activated by Single-Strand DNA Binding Protein in Mycobacteria

**DOI:** 10.1128/JB.00608-20

**Published:** 2021-01-25

**Authors:** Jemila C. Kester, Olga Kandror, Tatos Akopian, Michael R. Chase, Junhao Zhu, Eric J. Rubin, Alfred L. Goldberg, Sarah M. Fortune

**Affiliations:** a Department of Immunology and Infectious Diseases, Harvard TH Chan School of Public Health, Boston, Massachusetts, USA; b Department of Cell Biology, Harvard Medical School, Boston, Massachusetts, USA; Rutgers University-Robert Wood Johnson Medical School

**Keywords:** DNA replication, mycobacteria, cell cycle, protein degradation

## Abstract

The ClpP1P2 proteolytic complex is essential in Mycobacterium tuberculosis. Proteolysis by ClpP1P2 requires an associated ATPase, either ClpX or ClpC1. Here, we sought to define the unique contributions of the ClpX ATPase to mycobacterial growth. We formally demonstrated that ClpX is essential for mycobacterial growth, and to understand its essential functions, we identified ClpX-His-interacting proteins by pulldown and tandem mass spectrometry. We found an unexpected association between ClpX and proteins involved in DNA replication, and we confirm a physical association between ClpX and the essential DNA maintenance protein single-stranded-DNA binding protein (SSB). Purified SSB is not degraded by ClpXP1P2; instead, SSB enhances ATP hydrolysis by ClpX and degradation of the model substrate GFP-SsrA by ClpXP1P2. This activation of ClpX is mediated by the C-terminal tail of SSB, which had been implicated in the activation of other ATPases associated with DNA replication. Consistent with the predicted interactions, depletion of *clpX* transcript perturbs DNA replication. These data reveal that ClpX participates in DNA replication and identify the first activator of ClpX in mycobacteria.

**IMPORTANCE** Tuberculosis, caused by Mycobacterium tuberculosis, imposes a major global health burden, surpassing HIV and malaria in annual deaths. The ClpP1P2 proteolytic complex and its cofactor ClpX are attractive drug targets, but their precise cellular functions are unclear. This work confirms ClpX’s essentiality and describes a novel interaction between ClpX and SSB, a component of the DNA replication machinery. Further, we demonstrate that a loss of ClpX is sufficient to interrupt DNA replication, suggesting that the ClpX-SSB complex may play a role in DNA replication in mycobacteria.

## Abstract (precis)

Tuberculosis, caused by Mycobacterium tuberculosis, imposes a major global health burden, surpassing HIV and malaria in annual deaths. The ClpP1P2 proteolytic complex and its cofactor ClpX are attractive drug targets, but their precise cellular functions are unclear.

## INTRODUCTION

ClpX is a hexameric ATPase, a member of the family of ATPases associated with diverse cellular activities (AAA+), which are responsible for many essential cellular functions across domains, including DNA replication and maintenance and protein homeostasis. ClpX functions in ATP-dependent protein degradation by the ClpP proteolytic complex (1). ClpX selectively binds to, unfolds, and translocates targeted proteins into the Clp protease complex for degradation to small peptides. One of the best-characterized functions of bacterial ClpXP is degradation of SsrA-tagged proteins in the SsrA-tmRNA system (reviewed in reference 1). In some species, ClpX has also been shown to perform additional chaperone functions (2, 3). In most bacteria, the key players of this pathway—ClpX (ATPase), ClpP (protease), SmpB (substrate adaptor), and *ssrA* (transfer-messenger RNA [tmRNA])—are not essential for growth in rich medium but are required for growth under stress conditions (reviewed in reference 4). One exception to this majority is Caulobacter crescentus, where ClpXP is essential for growth because it regulates the initiation and elongation steps in DNA replication (5–7).

Mycobacterium tuberculosis encodes two ClpP proteins, ClpP1 and ClpP2, which form a two-ring, 14-subunit proteolytic complex (8). As in C. crescentus, both ClpP1 and ClpP2 are essential for growth in mycobacteria (9), which appears to be due, at least in part, to the ClpP1P2 complex’s role in the degradation of the essential transcription factor WhiB1 (10). In addition, the mycobacterial gene *clpX* is predicted to be essential for growth in culture by high-density transposon insertion analysis (11). However, this essentiality has not been independently tested, nor has the required function(s) of ClpX protein in mycobacteria been identified.

It has been proposed that ClpX is essential because it regulates cell division (12). Evidence from Escherichia coli and Bacillus subtilis, where the ClpXP complex is not essential, suggests that ClpXP regulates cell division through interaction with the tubulin homolog, FtsZ, although this interaction is not essential and other proteases also can catalyze FtsZ degradation (3, 13–15). In mycobacteria, depletion of the *clpX* transcript by antisense RNA and overproduction of a truncated ClpX protein inhibit cell division. Therefore, it was proposed that ClpX also modulates cell division through a similar interaction with FtsZ (12). However, this model has not been experimentally tested, nor is it clear whether ClpX mediates other critical functions in the cell.

Therefore, we sought to assess the essential functions of ClpX by identifying the proteins with which it interacts. These data reveal that ClpX interacts with key members of the replication machinery, including single-stranded-DNA binding protein (SSB). We biochemically defined an unexpected regulatory interaction in which SSB functions as a ClpX activator. Furthermore, we found that depletion of *clpX* results in failure to successfully complete DNA replication *in vivo*. Our work thus demonstrates that ClpX plays an important role in the DNA replication phase of the mycobacterial cell cycle and its activity can be regulated by SSB.

## RESULTS

### ClpX is essential for growth in rich medium.

**FIG 1:**
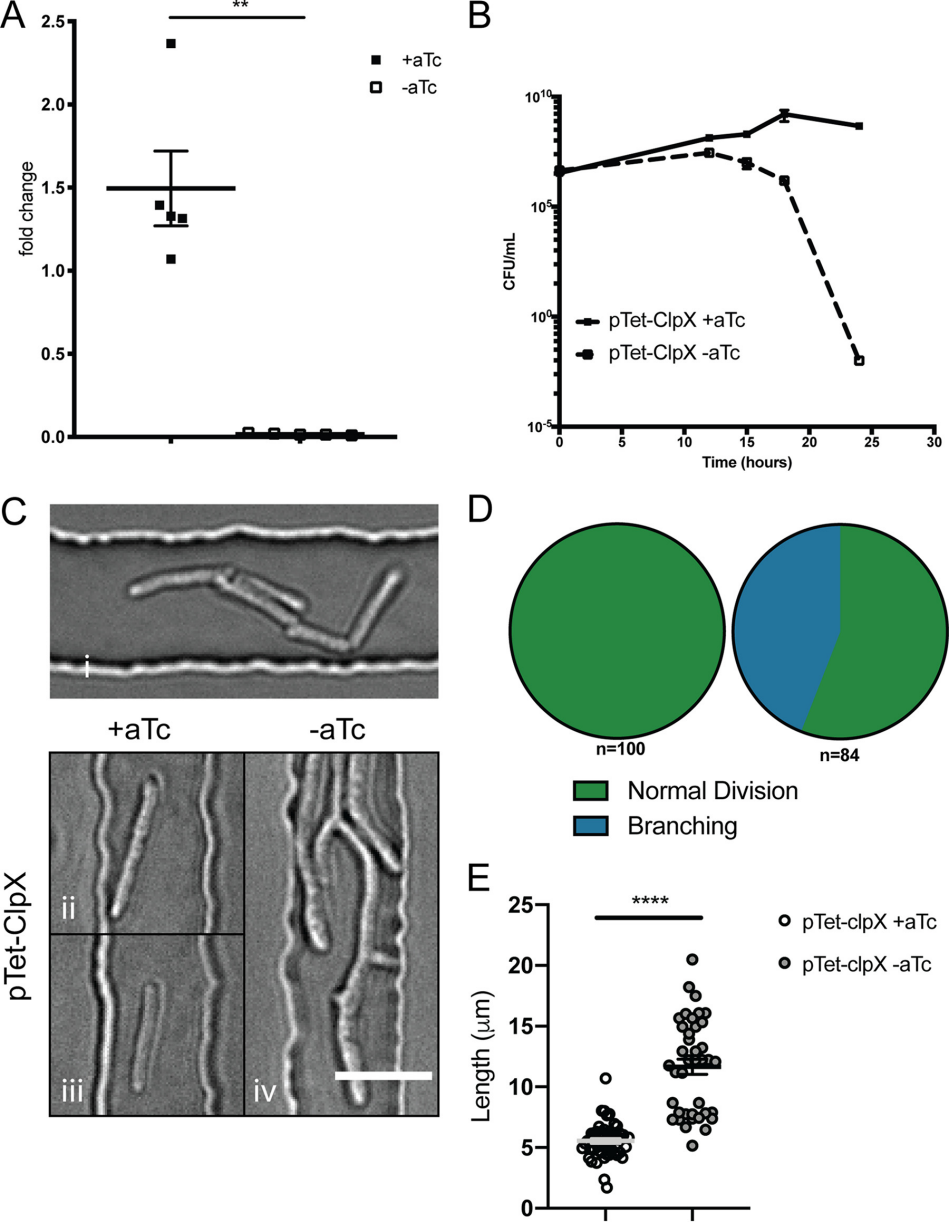
ClpX is essential for cell cycle completion. (A) Quantitative RT-PCR of *clpX* transcript from cultures of M. smegmatis carrying pTet_ON_-clpX in the presence or absence of inducer (+aTc and −aTc) at 24 h. Data are fold change relative to *sigA* (means and standard errors of the means [SEM]; *n* = 5 per condition). **, *P* = 0.0079 by Mann-Whitney. (B) Growth curves of M. smegmatis carrying pTet_ON_-clpX in the presence or absence of inducer (+aTc and −aTc). Data are means and SEM; *n* = 3 per condition. (C) Representative images of wild-type cells (i), isotype control cells (ii and iii), and cells depleted of *clpX* (iv). Bar, 5 μm. (D) Counts of division events in M. smegmatis carrying pTet_ON_-clpX in the presence or absence of inducer. Data show the ultimate fate of each cell, either a division event or branched cell. (E) Quantification of cell lengths for M. smegmatis carrying pTet_ON_-clpX in the presence or absence of inducer (+aTc and −aTc). Length of cells were measured using ImageJ. All cells are plotted (means and SEM). Significance was determined by a Mann-Whitney test. ****, *P* < 0.0001.

While the *clpX* gene is predicted to be essential for growth in mycobacteria by high-density transposon mutagenesis (11), this essentiality has not been experimentally tested. Therefore, using Mycobacterium smegmatis, a nonpathogenic relative of M. tuberculosis, we generated a strain in which the expression of *clpX* is regulated by a tetracycline-inducible promoter (Msm-pTet-clpX). With removal of anhydrous tetracycline (aTc), depletion of *clpX* transcript levels (Fig. 1A) resulted in a decrease in CFU below the limit of detection within 24 h (Fig. 1B). Phenotypically, depletion of *clpX* led to filamentation and branching (Fig. 1C to E), prior to cell death. Thus, ClpX is essential for both growth and viability.

### ClpX interacts with DNA replication machinery *in vivo*.

To understand the basis for branching, filamentation, and death, we sought to identify ClpX-interacting proteins. We overproduced an N-terminally 6×His-tagged ClpX (His_6_ClpX) from M. tuberculosis in M. smegmatis (alignment shown in Fig. S1 in the supplemental material). We lysed cells and pulled down His_6_ClpX-associated proteins. To prevent degradation—and thus obfuscation—of substrates by ClpXP1P2, we performed this entire assay in the presence of the nonhydrolyzable ATP analog ATPγS. Due to the inability of ATPγS to be hydrolyzed, it is able to lock ClpX in the substrate binding conformation and prevent substrate translocation (a process that requires ATP hydrolysis). Eluted proteins were then identified via tandem mass spectrometry and quantified via spectral counting.

**FIG 2:**
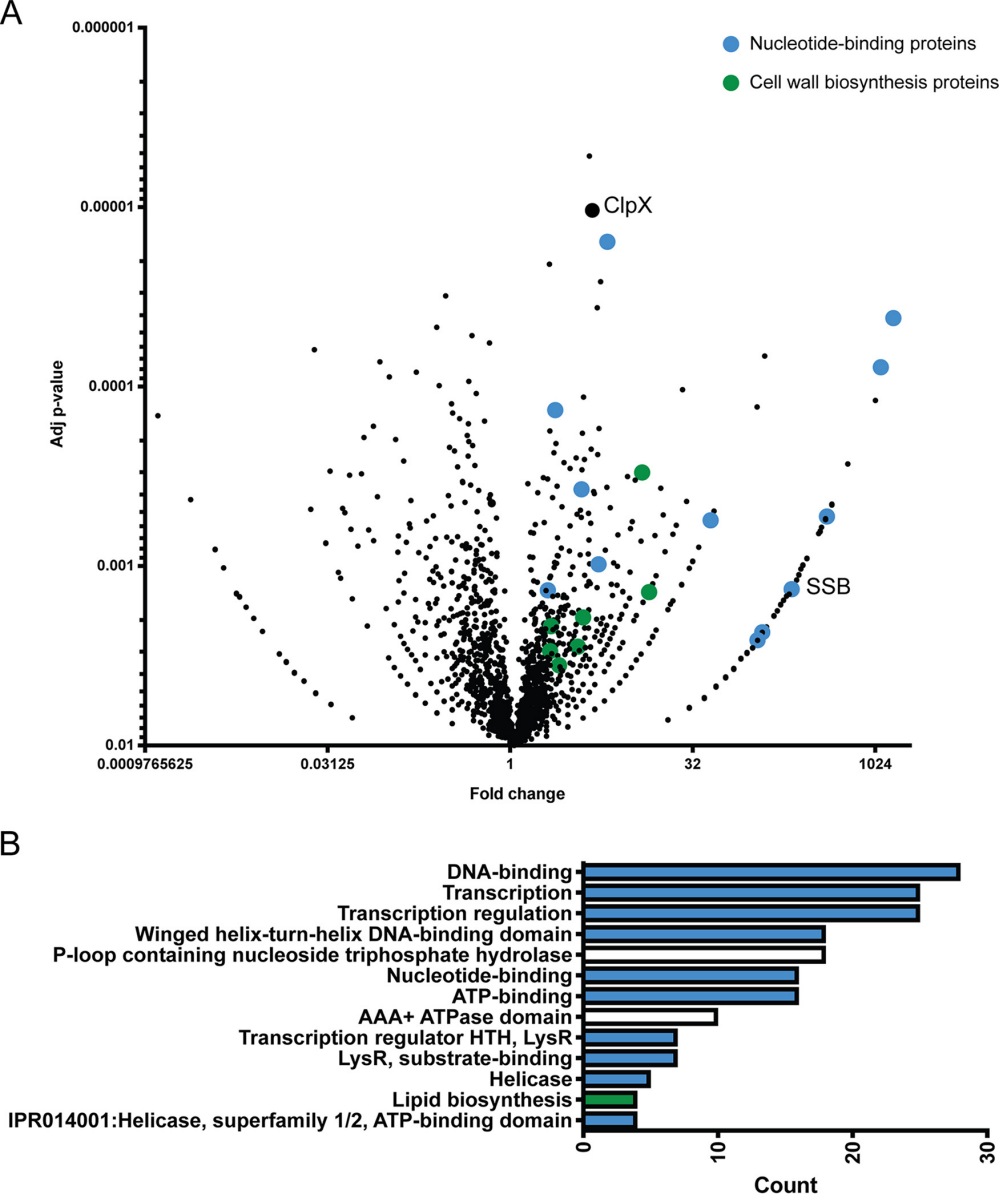
Identification of interactors of ClpX. (A) Volcano plot of total spectral counts as identified by MS/MS. Adjusted *P* values (determined by a G test with multiple-comparison correction) and fold changes are shown. Representative nucleotide-related proteins are indicated by blue highlighting. Representative cell wall synthesis or cell division proteins are indicated by green highlighting (proteins are listed in Table S2). (B) KEGG-based enrichment analysis with significance cutoffs of a fold change of ≥3.5 and an FDR of <0.05. Blue indicates KEGG pathways related to nucleotide binding. Green indicates KEGG pathways related to cell wall synthesis or cell division.

We identified 188 proteins that were significantly more abundant in the pulldowns from His_6_ClpX relative to controls, which we considered high-confidence putative ClpX-interacting proteins (Fig. 2A; Table S1). Previous work had suggested a direct role for ClpX in cell division through an interaction with the key cell division protein FtsZ (12). Consistent with this prior finding, we identified statistically significant associations with known cell wall elongation and cell division proteins (Fig. 2A; Table S2). However, we did not find FtsZ significantly associated with ClpX using this method.

To determine whether ClpX interacted preferentially with specific functional classes of proteins, we performed gene ontology enrichment analysis using KEGG pathway terms. This approach indicated that the ClpX interacting proteins were enriched for proteins involved in DNA replication, DNA repair, and transcription (Fig. 2B). Among the DNA replication and repair proteins identified, we found evidence of an association between ClpX and members of the replication machinery, including DnaB, where by mass spectrometry we identified peptides from DnaB exteins and, interestingly, from a natural intein contained within DnaB (Fig. S2). ClpX also coeluted with ParB, SSB, and key mediators of several DNA repair pathways, including RecA, RecC, RecD (homologous recombination), RadA (DNA repair protein), RuvC (Holliday junction resolvase), and UvrD (nucleotide excision repair) (16). Interestingly, recently published work (17) also describes an interaction between ClpX and replication machinery in mycobacteria.

### SSB activates ClpX ATPase activity and ClpXP1P2 protein degradation.

**FIG 3:**
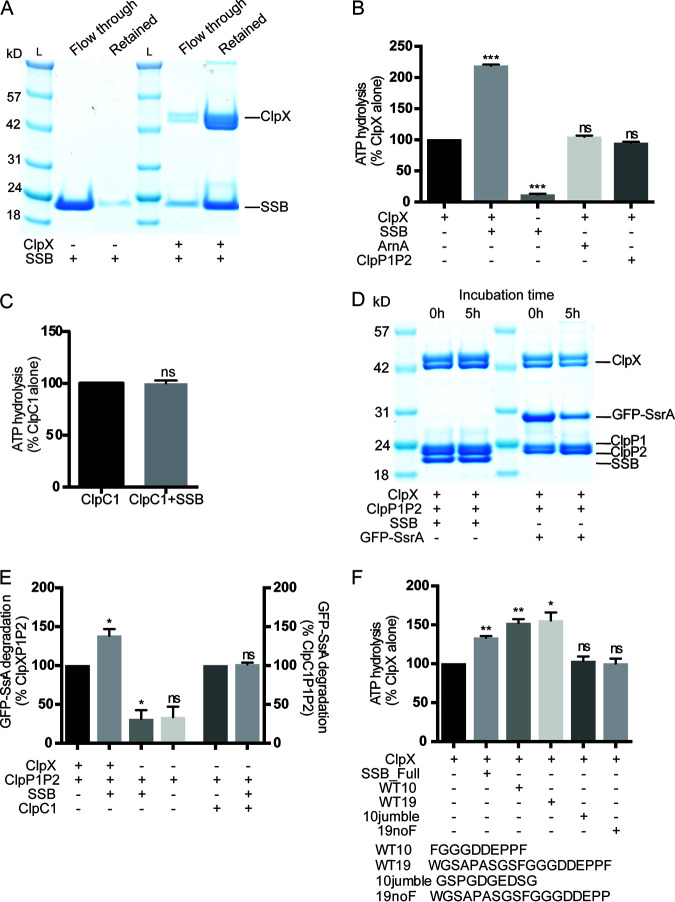
SSB activates ClpX and ClpXP1P2 via its C-terminal sequence. (A) SDS-PAGE gel of proteins retained on the filter or recovered in the eluate (flowthrough) in a filtration assay. (B) Rate of ATP hydrolysis for full-length proteins. Data are percent hydrolysis with ClpX alone. (C) Rate of ATP hydrolysis for full-length proteins. Data are percent hydrolysis with ClpC1 alone. (D) Degradation extent for SSB or GFP-SsrA at time zero or after 5 h of incubation with ClpXP1P2 at 37°C. (E) Rate of GFP-SsrA degradation. Data are percent degradation with ClpXP1P2 (left) or ClpC1P1P2 (right). (F) Rate of ATP hydrolysis for full-length SSB and short peptides. Data are percent hydrolysis with ClpX alone. Peptide sequences are listed beneath the graph. Data are representative of three biological replicates. *P* values are based on three technical replicates and are internally controlled. *, *P* < 0.05; **, *P* < 0.01; ***, *P* <0 .001; ns, not significant.

To test the hypothesis that ClpX associates with DNA-interacting proteins, we focused on single-stranded-DNA binding protein (SSB), which was highly enriched in our pulldown (Fig. 2A). SSB is an essential protein intricately involved in DNA replication and DNA repair (18). To confirm the physical interaction of SSB and ClpX, we used size-based filtration to separate free SSB from ClpX-complex-bound SSB. We employed this method using M. tuberculosis proteins purified from E. coli to determine if purified SSB and ClpX form a stable complex; if they do, SSB should remain on a 300-kDa filter with ClpX due to the increased size relative to isolated SSB. As expected, isolated SSB was recovered in the flowthrough from a 300-kDa cutoff filter, consistent with its molecular weight of 17 kDa (Fig. 3A), while isolated hexameric ClpX was retained on the filter, consistent with hexamer formation where the predicted molecular weight of the hexamer is ∼300 kDa. When ClpX and SSB were combined prior to filtration, SSB was retained on the filter together with ClpX (Fig. 3A). These data are consistent with a specific interaction between ClpX and SSB.

To define further the nature of the interaction between SSB and ClpX, we sought to determine whether the association with SSB alters ClpX activity. Again using M. tuberculosis proteins, we measured the rate of ATP hydrolysis by ClpX, establishing a dose-response curve demonstrating that ATP hydrolysis increases with increasing concentrations of SSB (Fig. S3). We then measured ATP hydrolysis in the presence or absence of a 10× molar excess of SSB using an enzyme-linked pyruvate kinase and lactic dehydrogenase (PK/LDH) assay (8, 19). SSB increased ClpX’s rate of ATP hydrolysis by 100% (Fig. 3B). An unrelated protein, ArnA, prepared in parallel, did not stimulate ATP hydrolysis by ClpX (Fig. 3B), nor did SSB stimulate ClpC1’s ATPase activity (Fig. 3C).

Interactors identified in our screen could include both substrates and regulators of ClpX. Both types of interactors would stimulate the ATPase activity of ClpX (20). To distinguish between these two possibilities, we asked whether SSB is degraded by the ClpXP1P2 proteolytic complex *in vitro* or *in vivo*. Incubation of SSB with pure ClpXP1P2 *in vitro* did not lead to SSB degradation, as determined by PAGE analysis, although the known ClpXP1P2 substrate green fluorescent protein (GFP)-SsrA was degraded under the same conditions (Fig. 3E). Aside from SsrA-tagged proteins, there are presently no known *in vitro* substrates of ClpXP1P2. Similarly, immunoblot analysis of cell lysates showed no change in the abundance of FLAG-tagged SSB following loss of ClpX from the cell compared to wild-type cells (Fig. S4). Thus, SSB does not appear to be a substrate of ClpXP1P2.

We therefore hypothesized that SSB may be a ClpX activator and tested whether SSB increased the rate of GFP-SsrA degradation by ClpXP1P2. We assayed GFP-SsrA degradation by ClpXP1P2 in the presence or absence of SSB using a cell-free fluorimetric assay described previously (19). No inhibition of GFP-SsrA degradation was observed in the presence of SSB, as would have been expected if both SSB and GFP-SsrA were substrates of ClpXP1P2. On the contrary, the rate of GFP-SsrA degradation increased 50% with the addition of SSB (Fig. 3D). This effect of SSB is specific to ClpXP1P2, as addition of SSB did not lead to degradation of GFP-SsrA by ClpC1P1P2 (Fig. 3D).

### SSB’s C terminus is sufficient and the terminal Phe is necessary to activate ClpX.

SSB has been shown to act as an organizer for chromatin-associated proteins (21) and to stimulate the activation of several ATPases involved in DNA replication and repair both in M. tuberculosis (22) and in other bacterial species (23, 24). SSB’s ability to activate proteins involved in DNA replication initiation and restart, DNA repair, and DNA recombination is mediated by its C-terminal tail (21). We therefore sought to determine whether activation of ClpX by SSB was also mediated by SSB’s C terminus. Based on a multiple sequence alignment of M. tuberculosis SSB to SSB in both E. coli and M. smegmatis (Fig. S5), we tested the ability of synthetic peptides representing either the terminal 10 or 19 residues (Fig. 3F; Fig. S5) to activate ClpX. Both the C-terminal 10- and 19-residue peptides increased ClpX ATP hydrolysis more than a scrambled version of the 10-mer (Fig. 3F). As with other ATPases activated by SSB, this modulation is dependent upon the terminal phenylalanine (Phe) (24), as peptides lacking the final Phe did not increase the rate of ATP hydrolysis above that of ClpX alone (Fig. 3F). The variation observed between ClpX hydrolysis in Fig. 3B and F is likely due to the fact the experiments were carried out at different times with different preparations of ClpX. To control for this natural variation, we ran each set of experiments in biological triplicate with at least 95% agreement. Thus, the final 10 residues of SSB are sufficient and the terminal phenylalanine is necessary for SSB to increase ClpX’s ATPase activity.

### ClpX is required for proper DNA replication.

**FIG 4:**
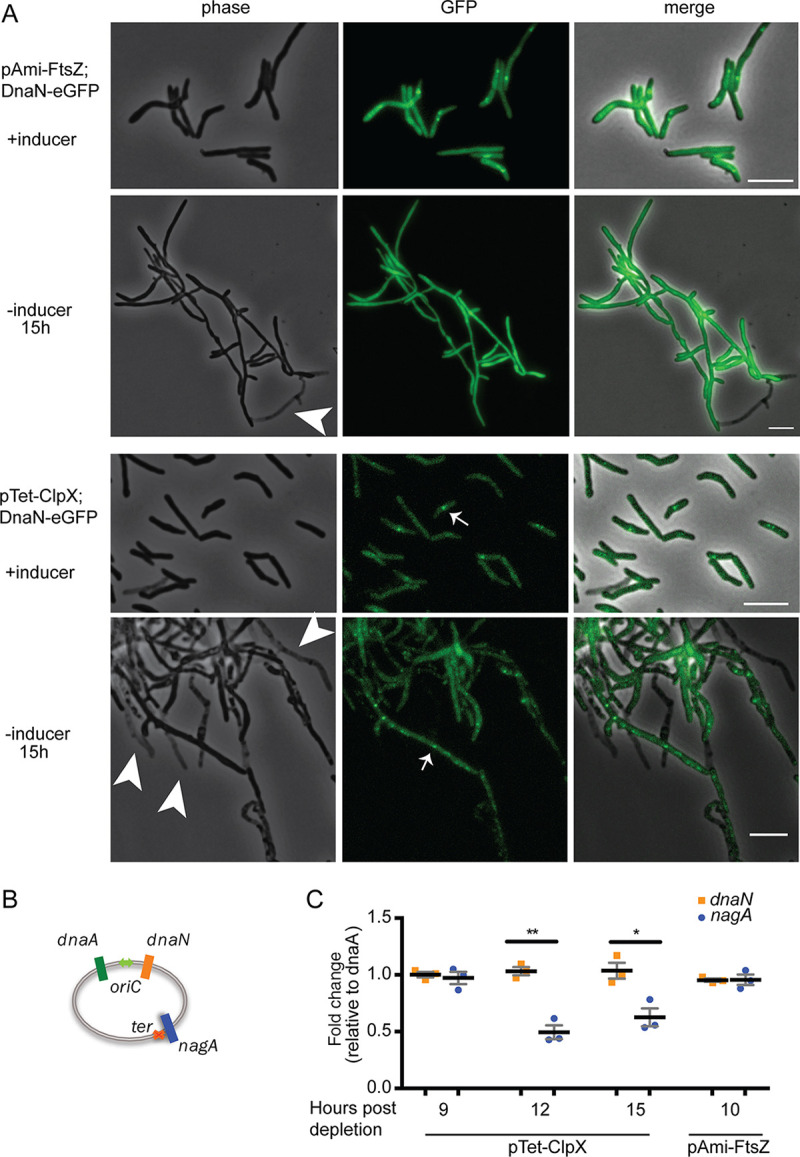
ClpX is required for proper DNA replication. (A) Representative images of DnaN-eGFP in cells of M. smegmatis carrying pTet_ON_-clpX or pAmi_ON_-ftsZ with or without inducer at 15 h after removal. Bar, 5 μm. Arrowheads indicate dead cells. Arrows indicate DnaN-eGFP foci. (B) Schematic of qPCR probes used in this assay. (C) qPCR data for the indicated cell types. Fold changes are shown relative to *dnaA* and inducer-containing controls. *P* values were determined by a *t* test. *, *P* < 0.05; **, *P* < 0.01.

We have shown that ClpX forms a stable complex with SSB, an essential member of the DNA replication machinery. To test the hypothesis that ClpX is directly involved in DNA replication, we assessed the impact of *clpX* transcript depletion on DNA replication *in vivo*. We used time-lapse microscopy to track DNA replication in real time in the presence and absence of ClpX. DNA replication initiation and termination are marked by the appearance and disappearance of the replicative helicase DnaN-eGFP (enhanced GFP) foci, respectively (25, 26). With *clpX* depletion, the number of DnaN-eGFP foci per cell increased relative to the number of foci found in control cells, suggesting an impairment in the completion of DNA replication (Fig. 4A).

Because loss of ClpX causes cell death that correlates with filamentation and branching and because ClpX has been suggested to interact with FtsZ, we asked whether inhibition of cell division through *ftsZ* transcript depletion would result in similar changes to the progression of DNA replication. Consistent with published data (27), *ftsZ* depletion led to cell death associated with filamentation and branching (Fig. 4A). However, the number of DnaN-eGFP foci per cell was not increased in *ftsZ*-depleted cells. Instead, *ftsZ* depletion resulted in a lack of visible DnaN-eGFP foci (Fig. 4A).

We therefore asked if *clpX* depletion perturbs progression of DNA replication (i.e., at a time after initiation but before termination of replication). To test this hypothesis, we created a quantitative PCR (qPCR)-based assay that assesses replication progression by quantifying the *ori*-flanking genes *dnaA* and *dnaN* and the *ter*-proximal gene *nagA* (Fig. 4B). Cells that fail to competently replicate their chromosome at a point after initiation should have a decreased ratio of *oriC* to *ter* regions relative to wild-type cells. We assessed replication progression in *clpX*-depleted cells compared to undepleted controls. There was a 50% reduction in *nagA* relative to *dnaA* after *clpX* depletion compared to undepleted cells, while the relative abundance of the *oriC*-flanking genes *dnaA* and *dnaN* did not change (Fig. 4C). As a control, we assessed the *oriC*-to-*ter* ratio in cells depleted of *ftsZ*, as these cells also filament and branch. The kill curve for depletion of *ftsZ* differs from that for depletion of *clpX*, with the former’s viability dropping significantly after 9 h after removal of inducer (27), while *clpX* depletion CFU do not drop below the level of detection until 18 h after removal of inducer. Therefore, we matched the dynamics of depletion of each strain, rather than the absolute time since removal of inducer. Depletion of *ftsZ* had no effect on the relative abundance of *nagA* compared to *dnaA* (Fig. 4C). These data suggest that ClpX is required for successful completion of mycobacterial DNA replication.

## DISCUSSION

Here, we tested the hypothesis that ClpX is essential and determined its interacting proteins. The identified interactors were consistent with our high-resolution phenotypic analysis, which implicated ClpX in DNA replication. Surprisingly, though successful, our approach did not identify a statistically significant association between ClpX and its known associated proteins, including ClpP1, ClpP2, the substrate adaptor SmpB, or FtsZ (12). These differences may reflect the fact that these associations are either less abundant or of lower affinity than ClpX’s associations with proteins involved in cell wall synthesis and DNA replication during logarithmic growth in rich medium.

To identify ClpX-associated proteins, we first attempted to create an ATPase-dead mutant of ClpX to trap substrates within the hexameric barrel, as was previously done in other species with nonessential Clp ATPases (28–30). However, the strain containing a Walker B mutant ClpX as a merodiploid (expressed as a second copy in a wild-type background) was not viable. This finding suggests that the mutant protein had a lethal dominant negative phenotype; either the mutant and wild-type monomers formed a nonfunctional complex, or the mutant monomers outcompeted the endogenous protein. Due to the lethality of the Walker B mutant ClpX, we instead used a chemically induced trap, locking ClpX in the open conformation with ATPγS.

We initially hypothesized that ClpXP1P2 has a regulatory function in DNA replication by degrading components of the replication machinery, including SSB and the replicative helicase DnaB intein 2, given their enrichment in our pulldown assay (210-fold and 69.5-fold, respectively [see Table S2 in the supplemental material]). However, the targeted validation studies suggest a more complex model in which SSB activates ClpX, which we presume then acts on key effectors which are yet to be identified and experimentally tested *in vivo*.

It is possible that DnaB is an important target of the ClpXP1P2-SSB association. Unfortunately, this hypothesis proved difficult to test *in vitro* due to the challenging nature of the mycobacterial DnaB protein. M. smegmatis and M. tuberculosis DnaB proteins contain two inteins and one intein, respectively (31). Inteins are protein introns, self-splicing protein components. Their autocatalytic ability makes intein-containing proteins impossible to purify. Peptides from the intein were identified in the analysis of the ClpX-interacting proteins, with a 69.5-fold enrichment (compared to a 3.88-fold enrichment across the entire peptide) (Table S2; Fig. S2). While we were unable to purify an intein-lost form of DnaB and did not find evidence that the spliced form of DnaB was a ClpX substrate *in vitro* (data not shown), we believe it likely that further experimentation may uncover a functional interaction between the ClpX-SSB complex and DnaB.

While typically presented as a DNA binding protein, SSB additionally organizes and activates DNA-associating proteins (21). The previously identified targets of SSB activation are canonical DNA replication and repair proteins (reviewed in reference 21). Interestingly, several of these, including RecA, RecC, RecD, RuvC, and UvrD, were identified as being associated with ClpX in our pulldown (Table S2). These interactions suggest a general model in which SSB coordinates the activities of multiple chromatin maintenance complexes aided by the chaperone activity of ClpX or degradation by the ClpXP1P2 complex. Our data suggest that SSB alone is not a substrate of ClpXP1P2. However, in its association with other proteins as a scaffold or in DNA replication proteins, SSB may become a substrate or may enhance the degradation of the associated proteins. Further studies will be needed to test this hypothesis.

## MATERIALS AND METHODS

### Bacterial culture conditions.

Strains used in this study are listed in Table 1. M. smegmatis mc^2^155 was cultured in Middlebrook 7H9 salts supplemented with 10% ADC (5:2:3 albumin-dextrose-catalase), 0.25% glycerol, and 0.05% Tween 80 or plated on Middlebrook 7H10 agar supplemented with ADC, 0.25% glycerol, and 0.05% Tween 80. All cultures were grown at 37°C, unless otherwise noted.

**TABLE 1:** M. smegmatis strains used in this work

Name	Description	Reference or source
mc^2^155	WT	ATCC
pTet-clpX	mc^2^155::Δ*clpX*; pTet-clpX	This study
His_6_ClpX	mc^2^155::pTet-His_6_-clpX	17
SSB-FLAG	pTet-clpX::pTb21-SSB-FLAG	This study
pTet-clpX; DnaN-eGFP	pTet-clpX::pTb21-DnaN-eGFP	This study
pAmi-ftsZ	mc^2^155::Δ*ftsZ*; pAmi-ftsZ	29
pAmi-ftsZ; DnaN-eGFP	pAmi-ftsZ::DnaN-eGFP	This study

For depletion of essential genes, *clp* family depletion line cultures were grown with the addition of anhydrotetracycline (aTc) at a final concentration of 100 ng/ml. Depletion was performed by washing logarithmically growing cell pellets in a volume of phosphate-buffered saline supplemented with 0.05% Tween 20 (PBS-T) equal to that of the original culture twice before resuspension in growth medium with or without aTc supplementation. Depletion of *ftsZ* was performed in the same manner, with the substitution of acetamide for aTc, at a final concentration of 0.2% by volume.

### Recombinant DNA and protein constructs.

MCtH::ptb21-FLAG-SSB and MCtH::ptb21-DnaN-eGFP were constructed using a customized Invitrogen multisite Gateway system created and generously donated by Christina Baer in Christopher Sassetti’s lab at University of Massachusetts Worcester Medical School, Worcester, MA. PCR was performed using Phusion high fidelity DNA polymerase (NEB catalog no. M0530). The gene of interest was then subcloned into the appropriate entry vector (pDO), and final constructs were made by combining the gene of interest in the appropriate pDO with entry vectors containing promoter and appropriate tag, and the destination vector. All genes in entry and destination vectors were sequenced to confirm that no mutations were introduced during PCR or subsequent cloning steps. All BP and LR reactions were performed using the BP Clonase II enzyme mix (Invitrogen catalog no. 11789-020) or LR Clonase II Plus enzyme mix (Invitrogen catalog no. 12538-120) in a 5-μl total volume overnight at room temperature. Next, protein digestion was performed by adding 1 μl proteinase K for 15 min at 37°C, and the entire 6-μl reaction mixture was electroporated into 15 μl DH5a electrocompetent cells prepared in our laboratory; cells were then plated on LB plates containing kanamycin (50 μg/ml) or the appropriate drug for the destination vector. Plasmids were transformed into M. smegmatis mc^2^155 cells made competent by three rounds of washing in 10% glycerol and electroporated, followed by 3 h recovery in 7H9 at 37°C. SSB peptides were synthesized by GenScript (Piscataway, NJ). Peptide sequences are as follows: WT10, FGGGDDEPPF; WT19, WGSAPASGSFGGGDDEPPF; 10jumble, GSPGDGEDSG; 19noF, WGSAPASGSFGGGDDEPP. To make the *clpX* depletion line, *clpX* was inserted into the L5 site of M. smegmatis mc^2^155 under the control of a tetracycline-inducible promoter using the nourseothricin (Nat) resistance marker. In the merodiploid, we then deleted *clpX* from the chromosome using recombineering and replacing it with a zeocin (Zeo) resistance marker. Doubly resistant (Nat and Zeo) M. smegmatis cells were then selected. To prevent a high rate of escape mutants, we added an episomal streptomycin-resistant plasmid containing several continuous *tetR* repeats (*tetR* plasmid was a gift from Kadamba Papavinasasundaram in Christopher Sassetti’s lab at University of Massachusetts Medical School, Worcester, MA). The *clpP1P2* depletion line used was a generous gift from Ravi Raju in Eric Rubin’s lab at the Harvard School of Public Health, Boston, MA. It was made using a tetracycline-inducible promoter, as described in their published work (9). The *ftsZ* depletion line was a gift from Malini Rajagopalan (27).

### Microscopy, time-lapse imaging, and image analysis. (i) Devices.

As previously described (32), microfluidic devices were made of polydimethylsiloxane (PDMS) bonded to no. 1.5 cover glass substrates using soft lithography techniques. Additional baking in a conventional oven at 65°C for at least 1 week aided in reducing background fluorescence.

### (ii) Microscope.

For images in Fig. 1, and as previously described (32), time-lapse images were acquired at 60× (Plan Apochromat objective; 1.42 numerical aperture [NA]) using a DeltaVision PersonalDV microscope with an automated stage enclosed in an environmental chamber warmed to 37°C. We used the InsightSSI solid-state illumination system (461 to 489 nm; Applied Precision, Inc.) to illuminate and a CoolSnap HQ2 camera (Photometric) to take pictures. We used the Ultimate Focus System (Applied Precision, Inc.) to maintain focus in time-lapse imaging. Images were acquired at the depletion time indicated. For images in Fig. 4, we used a Nikon Eclipse Ti-E inverted microscope equipped with the Nikon Perfect Focus System. Images were taken with a CFI Plan Apochromat Lambda oil objective (60×; 1.4 NA) on agar pad live mounts using a Lumencor Spectra X light engine with excitation/emission filters (fluorescein isothiocyanate [FITC]; 470 nm/503 nm). An Andor Zyla 4.2 sCMOS camera was used. The acquisition software was NIS Elements AR Advance, version 4.51.

### (iii) Image analysis.

Images were annotated using ImageJ (National Institutes of Health) with the ObjectJ plug-in (Norbert Vischer and Stelian Nastase, University of Amsterdam; http://simon.bio.uva.nl/objectj/index.html).

### Data representation and statistical analysis.

Prism 6.0a software (GraphPad Software, La Jolla, CA) was used to graph all data. Statistical tests of measurements were used from the Prism suite, as noted in the figure legends. Statistical analysis of mass spectrometry data was performed as described below.

### Pulldown and mass spectrometry.

Two technical replicates each of two biological replicates of logarithmically growing 1.5-liter cultures of ClpX-His and wild-type (WT) control cells were lysed with a French press in the presence of ATPγS-ATP (100:1) and DNase I at 4°C. Lysates were poured over nickel-nitrilotriacetic acid (Ni-NTA)–agarose beads (Thermo Fisher catalog no. R90101) overnight with shaking at 4°C. Elutions were performed with increasing concentrations of imidazole (0 to 200 mM in elution buffer). Eluates (100 mM and 500 mM) were collected, centrifuged at 14,000 × *g* and concentrated with trichloroacetic acid (TCA). The entire lysate was sent for tandem mass spectrometry (MS/MS) analysis to John Leszyk at University of Massachusetts Medical Center, Worcester, MA. Peptides were identified by nanoflow liquid chromatography (LC)-MS/MS on an Orbitrap (QExactive), and their relative abundances were quantitated based on spectral counts (the number of MS/MS events) and precursor intensity (MS1 integrated peak intensity) (33). We then used Mascot software in the Scaffold viewer to assign spectra to M. smegmatis proteins and quantify relative abundances of individual proteins between the samples. *P* values were determined by a G test with Benjamini-Hochberg correction for multiple sampling.

### KEGG pathway analysis.

Gene set enrichment analysis was performed using KEGG annotation (34) on the DAVID Bioinformatics Resources platform v6.8 from NIAID, NIH (35). Significance was set to a fold change of ≥3.5 and a false discovery rate (FDR) of <0.05.

### Biochemical validation. (i) Production and purification of proteins.

An N-terminally 6×His-tagged truncated form of ClpX (lacking the first 60 amino acids) and C-terminally 6×His-tagged ClpP1, ClpP2, and ClpC1 were produced from pTrc99 in E. coli BL21 Δ*clpXP* and purified as described previously (19). SSB from M. tuberculosis was a generous gift of Meindert Lamers at MRC, Cambridge, United Kingdom.

### (ii) ATPase assay (PK/LDH).

ATP hydrolysis was measured with the enzyme-linked assay using pyruvate kinase and lactic dehydrogenase (PK/LDH). Two micrograms of pure ClpC1 or ClpX and a 10× molar excess of SSB (where indicated) were mixed with 100 μl of assay buffer B containing 1 mM phosphoenolpyruvate (Sigma catalog no. 860077), 1 mM NADH (Sigma catalog no. N8129), 2 U of pyruvate kinase-lactic dehydrogenase, 4 mM MgCl_2_, and 1 mM ATP, and the ATPase activity was followed by measuring the oxidization of NADH to NAD spectrometrically at 340 nm. Measurements were performed in triplicate, which agreed within 5%.

### (iii) Proteinase assay.

ClpXP1P2 was assayed continuously in 96-well plates using the fluorescent protein substrate GFP-SsrA. To measure GFP-SsrA degradation by the ClpXP1P2 complex, each well contained 500 nM GFP-SsrA, 75 to 100 nM ClpP1P2 tetradecamer, 300 to 400 nM ClpX hexamer, and 2 mM Mg-ATP in 100 μl of buffer A (20 mM phosphate buffer [pH 7.6] with 100 mM KCl, 5% glycerol, and 2 mM benzoyl-Leu-Leu). GFP-SsrA fluorescence was measured at 509 nm (excitation at 440 nm).

### Protein analysis by immunoblotting.

Protein lysates were extracted using bead beating in FLAG or His buffer. Whole-cell lysates were run on NuPAGE 4 to 12% bis-Tris protein gels (Thermo Fisher catalog no. NP0322BOX). For FLAG immunoblotting, we used primary mouse anti-DYKDDDDK (FLAG epitope tag) antibody, clone 2EL-1B11 (EMD Millipore catalog no. MAB3118), at 1:500. For secondary blotting, we used a WesternBreeze anti-mouse antibody chromogenic kit (Thermo Fisher catalog no. WB7103) according to the manufacturer’s instructions. For a loading control, we used anti-GAPDH (Ga1R) loading control mouse monoclonal antibody from Pierce Chemical (catalog no. MA515738) at 1:5,000. For secondary blotting, we used goat anti-mouse IgG (heavy plus light chain [H+L]) secondary antibody conjugated to horseradish peroxidase (HRP; catalog no. 32430; Thermo Fisher) at 1:5,000.

### qPCR and qRT-PCR assays.

Genomic DNA extraction was performed using the lab’s phenol-chloroform method as previously described (36). Quantitative PCR (qPCR) was performed on 20 ng of genomic DNA (gDNA) using in-house primer sets. All primer sets were tested and matched for efficiency using a standard curve of known target concentration prior to use in this assay. Detection of product amplification was by iTRAQ Universal SYBR green supermix (Bio-Rad catalog no. 1725121) on an Applied Biosystems 7300 real-time PCR system. Expression values are a product of the ΔΔ*C_T_* method, normalized to *dnaA* and using time zero as the control. RNA was extracted using the standard TRIzol (Thermo Fisher catalog no. 15596026) method with the addition of 45 s and 30 s bead-beating in a FastPrep24 homogenizer (MP Bio, Santa Ana, CA) to aid in lysis. DNA was removed by the addition of 10 U DNase Turbo (Ambion catalog no. AM2238) for 1 h and purified with RNeasy (Qiagen catalog no. 74104) according to the manufacturer’s instructions. cDNA synthesis was performed with a SuperScript III first-strand synthesis kit (Thermo Fisher catalog no. 18080051) and random hexamers, according to the product manual. Quantitative reverse transcriptase PCR (qRT-PCR) was performed as described for qPCR above, using cDNA instead of gDNA. Expression values are a product of the ΔΔ*C_T_* method, normalized to *sigA* and using a no-aTc control.

### Data availability.

The proteomics data set generated in this work is available under the accession number MSV000084267 on MassIVE at https://massive.ucsd.edu/ProteoSAFe/dataset.jsp?task=64c03fd404724284ab93fa972a2d9b69.

## Supplementary Material

Supplemental file 1

Supplemental file 2
