# Supplementary material for: ClpX Is Essential and Activated by Single-Strand DNA Binding Protein in Mycobacteria
Source: J Bacteriol. 2021 Jan 25;203(4):e00608-20. doi: 10.1128/JB.00608-20 (PMC7847540; doi:10.1128/JB.00608-20)
Supplement: Supplemental file 2 [file JB.00608-20-s0002.pdf]

Supplementary Figures

Table S1: Complete list of proteins identified by MS/MS and their relative abundances.
This table is too large for inclusion here. It is attached as a separate document.

Table S2: Representative list of select proteins from ClpX pulldown involved in cell wall biosynthesis and nucleotide binding.

Protein Name	adj p	Ratio	Cell Function
MamA DNA methylase	7.79E-05	1140.00	nucleotide binding
RadA, DNA repair protein	5.30E-04	410.00	nucleotide binding
SSB, ssDNA-binding protein	1.34E-03	210.00	nucleotide binding
RuvC, Crossover (Halliday) junction endodeoxyribonuclease	2.34E-03	120.00	nucleotide binding
RecD, exonuclease V (Alpha chain)	2.59E-03	110.00	nucleotide binding
RecC, exonuclease V (Gamma chain)	5.56E-04	45.00	nucleotide binding
UvrD/REP helicase	9.76E-04	5.36	nucleotide binding
DnaB, Replicative DNA helicase	3.74E-04	3.88	nucleotide binding
RecA	1.35E-04	2.36	nucleotide binding
hypothetical septum_form (ZipA-like)	6.07E-04	370.00	division
PknF, serine/threonine-protein kinase	1.94E-03	4.00	division
PknB, serine/threonine-protein kinase	2.17E-03	2.17	division
MurB, UDP-N-acetylenolpyruvoylglucosamine reductase	1.40E-03	14.00	divi/elongasome
PonA2	3.01E-04	12.29	divi/elongasome
MurM ligase, UDP-N-acetylmuramyl tripeptide synthase	1.00E-03	7.67	divi/elongasome
MurE, UDP-N-acetylmuramoylalanyl-D-glutamate-2,6-diaminopimelate	2.81E-03	3.63	divi/elongasome
bifunctional penicillin-binding protein 1A/1B PonA1	2.98E-03	2.14	divi/elongasome

```
Mtuberculosis      --MARIGDGGDLLKCSFCGKSQKQVKKLIAGPGVYICDECIDLCNEIIEEELADADDVK-
Msmegmatis         --MARIGDGGDLLKCSFCGKSQKQVKKLIAGPGVYICDECIDLCNEIIEEELADADDVK-
Ecoli              MTDKRKDGSGKLLYCSFCGKSQHEVRKLIAGPSVYICDECVDLCNDIIREEIKEVAPHRE
                       * ...*.** ********::*:******.*******:****:**.**: :.   :

Mtuberculosis      LDELPKPAEIREFLEGYVIGQDTAKRTLAVAVYNHYKRIQAGEKGRDSRCEPVELTKSNI
Msmegmatis         LDELPKPAEIREFLEGYVIGQDSAKRTLAVAVYNHYKRIQAGEKSRDSRSEPVELTKSNI
Ecoli              RSALPTPHEIRNHLDDYVIGQEQAKKVLAVAVYNHYKRLRNGDTSNG-----VELGKSNI
                    . **.* ***:.*:.*****: **:.***********:: *:....      *** ****

Mtuberculosis      LMLGPTGCGKTYLAQTLAKMLNVPFAIADATALTEAGYVGEDVENILLKLIQAADYDVKR
Msmegmatis         LMLGPTGCGKTYLAQTLAKMLNVPFAIADATALTEAGYVGEDVENILLKLIQAADYDVKR
Ecoli              LLIGPTGSGKTLLAETLARLLDVPFTMADATTLTEAGYVGEDVENIIQKLLQKCDYDVQK
                   *::****.*** **:***::*:***::****:**************: **:* .****::

Mtuberculosis      AETGIIYIDEVDKIARKSENPSITRDVSGEGVQQALLKILEGTQASVPPQGGRKHPHQEF
Msmegmatis         AETGIIYIDEVDKIARKSENPSITRDVSGEGVQQALLKILEGTQASVPPQGGRKHPHQEF
Ecoli              AQRGIVYIDEIDKISRKSDNPSITRDVSGEGVQQALLKLIEGTVAAVPPQGGRKHPQQEF
                   *: **:****:***:***:*******************::*** *:*********:***

Mtuberculosis      IQIDTTNVLFIVAGAFAGLEKIIYERVGK-RGLGFGAEVRSKAEIDTT-DHFADVMPEDL
Msmegmatis         IQIDTTNVLFIVAGAFAGLEKIVSDRVGK-RGLGFGAEVRSKAEIDTQ-DHFAEVMPEDL
Ecoli              LQVDTSKILFICGGAFAGLDKVISHRVETGSGIGFGATVKAKSDKASEGELLAQVEPEDL
                   :*:**:::*** .******:*:: .** .   *:**** *::*::  :  : :*:* ****

Mtuberculosis      IKFGLIPEFIGRLPVVASVTNLDKESLVKILSEPKNALVKQYIRLFEMDGVELEFTDDAL
Msmegmatis         IKFGLIPEFIGRLPVVASVTNLDKASLVKILSEPKNALVKQYVRLFEMDGVELEFTEEAL
Ecoli              IKFGLIPEFIGRLPVVATLNELSEEALIQILKEPKNALTKQYQALFNLEGVDLEFRDEAL
                   *****************::.:*.: :*::**.******.***  **:::**:*** ::**

Mtuberculosis      EAIADQAIHRGTGARGLRAIMEEVLLPVMYDIPSRDDVAKVVVTKETVQDNVLPTIVPRK
Msmegmatis         EAIADQAIHRGTGARGLRAIMEEVLLPVMYDIPSRDDVAKVVVTKETVQDNVLPTIVPRK
Ecoli              DAIAKKAMARKTGARGLRSIVEAALLDTMYDLPSMEDVEKVVIDESVIDGQSKPLLIYGK
                   :***.:*: * *******:*:* .** .***:** :** ***: :..::.:  * ::  *

Mtuberculosis      PSRSERRDKSA
Msmegmatis         PSRSERRDKSA
Ecoli              PEAQQASGE--
```

Figure S1: Multiple sequence alignment of ClpX. Alignment by CLUSTAL 2.1 of ClpX sequences from *M. tuberculosis*, *M. smegmatis*, and *E. coli*. Residue homology is indicated as [*] identical; [:] conserved; [.] semi-conserved; or [ ] not conserved.

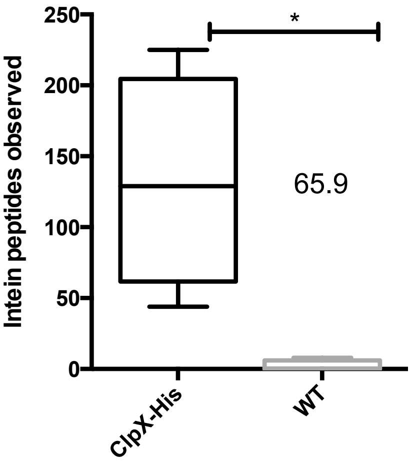

Figure S2: Intein2 from $DnaB_{SM}$ abundance in pulldowns. Total peptides identified by LC-MS/MS from His_6ClpX and wildtype cultures, as shown in Table S1. The increase in abundance is indicated. p-values by t-test: *<0.05.

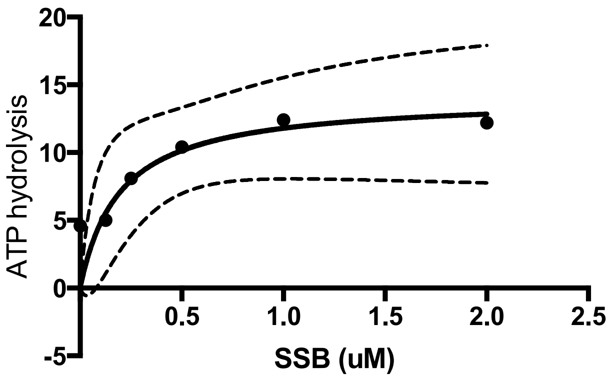

Figure S3: Dose-response curve. A dose-response curve was created for the impact of SSB concentration on ClpX ATP hydrolysis using the PK/LDH assay. Hydrolysis rate is shown on the the y-axis, while SSB concentration in micromolar units is shown on the x-axis.

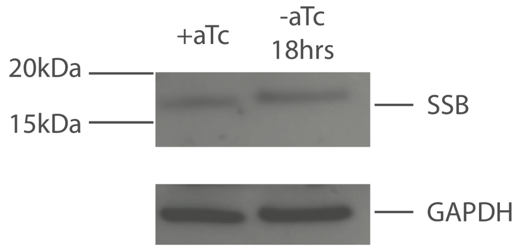

Figure S4: SSB does not accumulate with *clpX* depletion *in vivo*. Anti-FLAG immunoblot for SSB-FLAG (upper) with loading control anti-GAPDH (lower). Whole lysate from Msm_pTet$_{ON}$-clpX cells +/- inducer for 18hrs.

```
Mtuberculosis      --MAGDTTITIVGNLTADPELRFTPSGAAVANFTVASTPRIYDRQTGEWKDGEALFLRCN
Msmegmatis         --MAGDTTITVVGNLTADPELRFTPSGAAVANFTVASTPRMFDRQSGEWKDGEALFLRCN
ECOLI              MASRGVNKVILVGNLGQDPEVRYMPNGGAVANITLATSESWRDKATGEMK-EQTEWHRVV
                       * ..: :****  ***:*: *.*.****:*:*::    *: :** *  :: : *

Mtuberculosis      IWREAAENVAESLTRGARVIVSGRLKQRSFETREGEKRTVIEVEVDEIGPSLRYATAKVN
Msmegmatis         IWREAAENVAESLTRGSRVIVTGRLKQRSFETREGEKRTVVEVEVDEIGPSLRYATAKVN
ECOLI              LFGKLAEVASEYLRKGSQVYIEGQLRTRKWTDQSGQDRYTTEVVVNVGGTMQMLGGRQGG
                   :: : ** .:* * :*::* : *:*: *.:  :.*:.* . ** *:  *.    .  : .

Mtuberculosis      KASRSGGFGSGSRP-APAQTSSASGDDPWG------------SAPASGSFGGGDDEPPF
Msmegmatis         KASRSGGGGGGFGSGGGGSRQSEPKDDPWG------------SAPASGSFSGADDEPPF
ECOLI              GAPAGGNIGGGQPQGGWGQPQQPQGGNQFSGGAQSRPQQSAPAAPSNEPPMDFDDDIPF
                    *. .*. *.*    . .. ..   .: :.            :**:. .  . **: **
```

Figure S5: Multiple sequence alignment of SSB. Alignment by CLUSTAL 2.1 of SSB sequences from *M. tuberculosis*, *M. smegmatis*, and *E. coli*. Peptide constructs: WT19 is underlined. WT10 is italicized. Terminal phenylalanine in teal. Residue homology is indicated as [*] identical; [:] conserved; [.] semi-conserved; or [ ] not conserved.
